# A Deep Insight Into Regulatory T Cell Metabolism in Renal Disease: Facts and Perspectives

**DOI:** 10.3389/fimmu.2022.826732

**Published:** 2022-02-17

**Authors:** Zhongyu Han, Kuai Ma, Hongxia Tao, Hongli Liu, Jiong Zhang, Xiyalatu Sai, Yunlong Li, Mingxuan Chi, Qing Nian, Linjiang Song, Chi Liu

**Affiliations:** ^1^ Department of Nephrology, Sichuan Academy of Medical Science and Sichuan Provincial People’s Hospital, Sichuan Renal Disease Clinical Research Center, University of Electronic Science and Technology of China, Chengdu, China; ^2^ Chinese Academy of Sciences Sichuan Translational Medicine Research Hospital, Chengdu, China; ^3^ Reproductive & Women–Children Hospital, School of Medical and Life Sciences, Chengdu University of Traditional Chinese Medicine, Chengdu, China; ^4^ Department of Nephrology, Osaka University Graduate School of Medicine, Osaka, Japan; ^5^ Affiliated Hospital of Inner Mongolia University for the Nationalities, Tongliao, China; ^6^ Department of Blood Transfusion Sicuan Provincial People’s Hospital, University of Electronic Science and Technology of China, Chengdu, China

**Keywords:** metabolic pathways, regulatory T cells, renal disease, tissue damage, immune homeostasis

## Abstract

Kidney disease encompasses a complex set of diseases that can aggravate or start systemic pathophysiological processes through their complex metabolic mechanisms and effects on body homoeostasis. The prevalence of kidney disease has increased dramatically over the last two decades. CD4^+^CD25^+^ regulatory T (Treg) cells that express the transcription factor forkhead box protein 3 (Foxp3) are critical for maintaining immune homeostasis and preventing autoimmune disease and tissue damage caused by excessive or unnecessary immune activation, including autoimmune kidney diseases. Recent studies have highlighted the critical role of metabolic reprogramming in controlling the plasticity, stability, and function of Treg cells. They are also likely to play a vital role in limiting kidney transplant rejection and potentially promoting transplant tolerance. Metabolic pathways, such as mitochondrial function, glycolysis, lipid synthesis, glutaminolysis, and mammalian target of rapamycin (mTOR) activation, are involved in the development of renal diseases by modulating the function and proliferation of Treg cells. Targeting metabolic pathways to alter Treg cells can offer a promising method for renal disease therapy. In this review, we provide a new perspective on the role of Treg cell metabolism in renal diseases by presenting the renal microenvironment、relevant metabolites of Treg cell metabolism, and the role of Treg cell metabolism in various kidney diseases.

## Introduction

**Figure 1:**
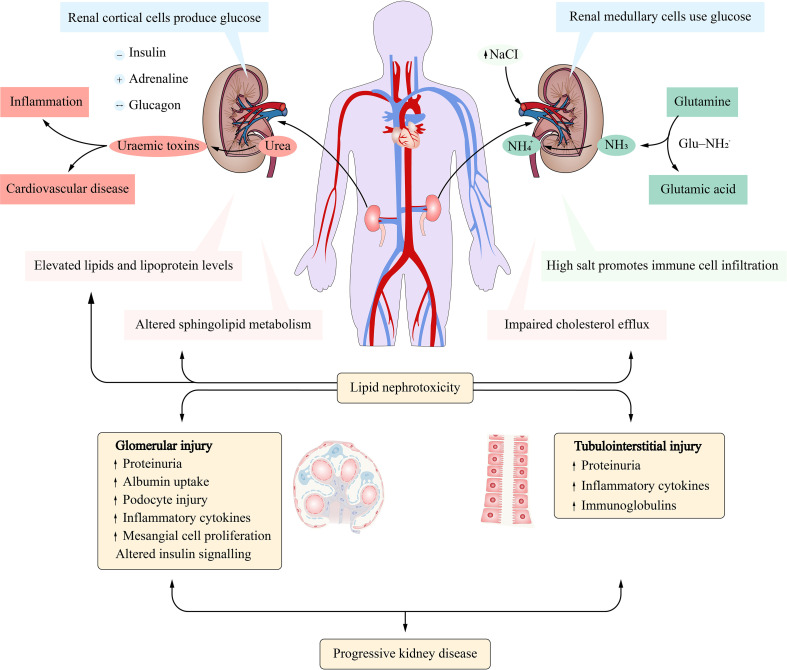
Metabolism of substances in the kidney. Cortical cells undergo gluconeogenesis while medullary cells metabolize glucose. Glutamine is extracted from renal tubular cells and used to produce ammonia (NH3). High levels of urea cause the kidneys to produce uremic toxins. Lipid nephrotoxicity could damage the structure and function of the glomerulus and tubules.

The kidney is an important organ for excreting metabolic waste and maintaining internal environmental stability and plays an extremely important role in metabolic activities (1) (**Figure 1**). Treg cells are typical CD4^+^ cells that constitutively express high levels of the interleukin-2 (IL-2) receptor CD25, along with the transcription factor Foxp3, which plays a central role in generating and maintaining Treg cell-specific gene expression by cooperating with other transcription factors, such as runt-related transcription factor 1 (RUNX1) and gata binding protein 3 (GATA3) (2).

**Figure 2:**
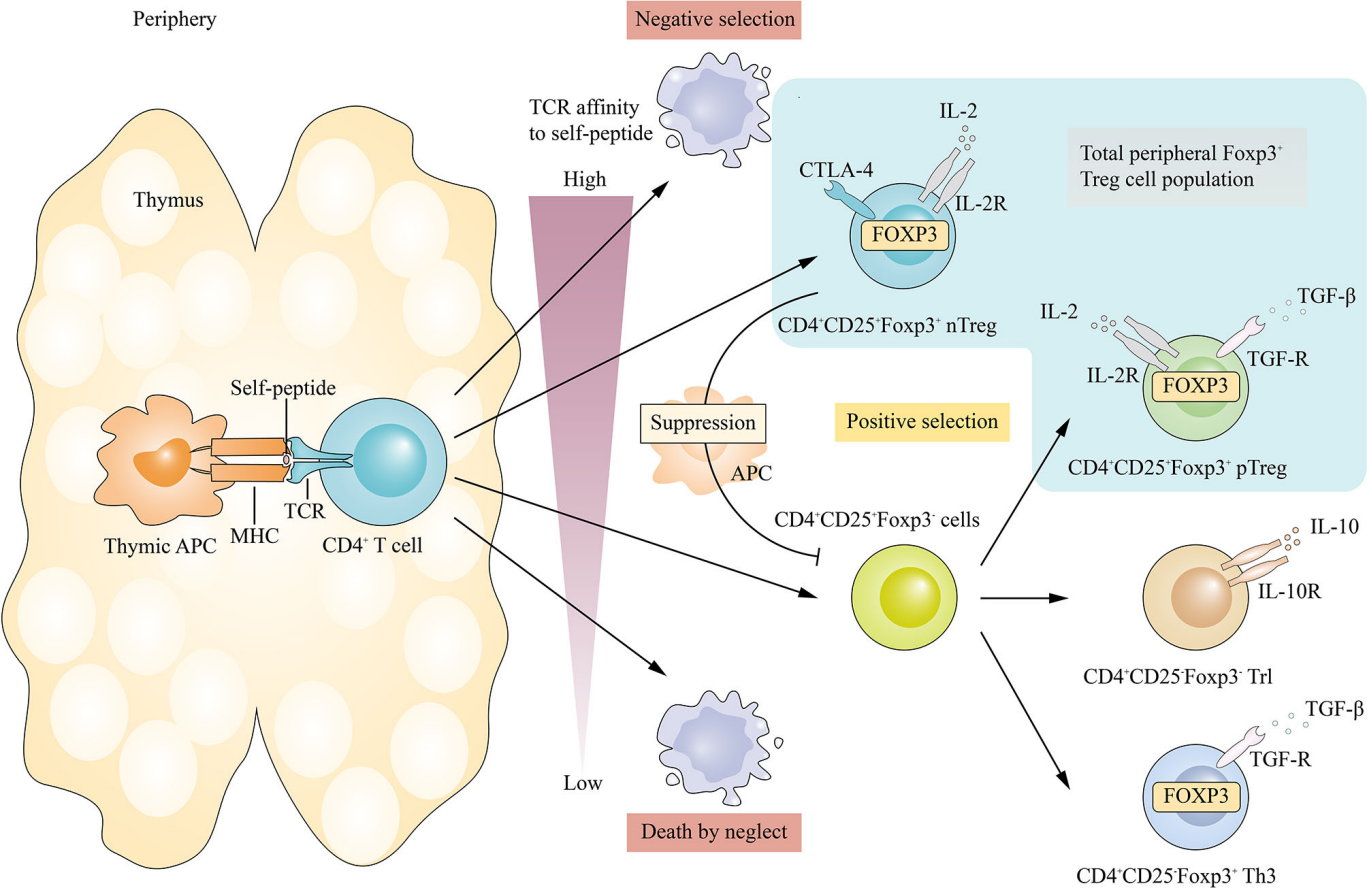
Differentiation of Treg cells *in vivo*. TTreg cell development is initiated by TCR signal transduction. CD4+CD8- thymocytes that bind with high affinity to their self- peptides-MHC complex are positively selected in the thymus. Immature T cells with low affinity for their own peptide-MHC complex are also positively selected and subsequently differentiated into different subtypes, including pTreg, Tr1 and Th3.

Treg cells *in vivo* can be divided into two types (3): thymus Treg cells (tTreg), which mature after positive and negative selection in the thymus and play an immunosuppressive role in peripheral blood and lymphoid tissues; and peripherally induced Treg (pTreg) cells, which originate from T cells after antigenic stimulation and are converted by inhibitory cytokines (**Figure 2**). Treg cells *in vitro* are induced by cytokines and other factors, often referred to as induced CD4^+^ T regulatory cells (iTreg). *In vitro* and *in vivo*, CD4^+^CD25^+^Foxp3^+^ Treg cells inhibited the activation, proliferation, and effector function of a wide range of immune cells, such as CD4^+^ and CD8^+^ T cells, natural killer (NK) cells, and NKT cells. They are indispensable for the maintenance of self-tolerance and immune homeostasis by inhibiting excessive or misdirected immune responses to foreign or autogenous targets (4).

It is important to note that phenotypic differences between tTreg cells and pTreg cells have not been clearly defined, which poses challenges in distinguishing the exact proportions of these two subpopulations in secondary lymphoid organs and non-lymphoid tissues. Studies have shown that neuropilin (Nrp-1) and Helios are highly expressed on tTreg in mice, but not on pTreg/iTreg cells (5, 6). Therefore, some researchers believe that tTreg cells and pTreg cells can be distinguished by Nrp-1 and Helios. However, this hypothesis has been controversial, especially when it comes to the distinction between human tTreg cells and pTreg cells (7, 8). Therefore, in the following review, we tried our best to use accurate classification to describe Treg cells, such as tTreg cells, pTreg cells, and iTreg cells. Where we were unable to distinguish the origins of the Treg cells from the original article, we have described the population studied using ‘Treg cell’ only.

Recently, increasing evidence has shown that Treg cells can take part in various renal diseases. Treg cells can play a negative regulatory role in kidney diseases and inhibit the immune response through direct cell contact or secretion of inhibitory cytokines (9). At the same time, kidney diseases, in turn, affect the function of Treg cells. For example, the number of Treg cells in patients with IgA nephritis is significantly reduced (10).

It is well known that renal disease is accompanied by significant changes in metabolic patterns (11), such as changes in glucose (12), amino acid (13), and lipid metabolism (14), which are essential for the activation and proliferation of Treg cells. Moreover, the metabolic pattern of Treg cells is also regulated by the metabolic state of nephropathy, and the type of nutrients used by Treg cells in nephropathy changes their differentiation, resulting in alterations in their phenotype and proportion. In addition to nutritional supply, the accumulated byproducts of renal metabolism significantly impair the immunosuppressive function of Treg cells, and the loss or functional deficiency of Treg cells affects the immune homeostasis of the kidney (15–18).

In the following sections, we will introduce renal microenvironment. Treg cell metabolism, the role of Treg cells in various renal diseases, and the importance of abnormalities in various metabolic pathways for the function of Treg cells, and will discuss the factors of abnormal metabolic pathways, which may be the goal of immunotherapy for related renal diseases.

## Renal Microenvironment

The kidney is the most important organ in the human urinary system, which undertakes the important mission of filtering metabolic waste, excreting them from the body, and reabsorbing various nutrients (19). The kidney can maintain the body fluid and electrolyte balance by the distal tubule of the collecting duct through the absorption and excretion of various ions (electrolytes) in the body, such as sodium ions, phosphorus ions, calcium ions, and magnesium ions (20), at the same time, discharge the vast majority of metabolic wastes produced by the human body (21), for example, urea nitrogen, creatinine, uric acid, etc., to prevent waste products accumulate in the body, causing various disorders.

The kidney is an organ with important functions and complex structures, which determines that there are many kinds of cells involved in the microenvironment of the kidney, including immune cells and intrinsic cells of the kidney. Cytokines, chemokines, adhesion molecules, and complement secreted by immune cells and intrinsic cells of the kidney in the local immune microenvironment of kidney tissue, which plays a great role in the occurrence of kidney metabolism and injury.

### Immune Cells and Intrinsic Cells of the Kidney

#### Macrophages

Macrophages are classified into M1 macrophages and M2 macrophages (22). In healthy kidney tissue, the main function of macrophages is to phagocytose and digest cell fragments and pathogens in the form of fixed cells or free cells, and to activate lymphocytes or other immune cells to respond to pathogens.

Studies have shown that macrophages play an important role in mediating immunopathology and tissue remodeling in non-renal disease and renal disease (23). In animal models, blocking macrophage recruitment and expression of inflammatory factors can prevent the progression of various kidney diseases (24). At the same time, the damaged kidney produces a large number of macrophages, which continuously infiltrate the kidney and produce pro-inflammatory cytokines, including TNF-α and IL-1β, to induce kidney inflammation (25). In addition, macrophages can produce oxygenated nitric oxide Complement components can directly damage renal cells and affect the formation of matrix and blood vessels by expressing matrix metalloproteinases and vasoactive peptides (26).

#### Mast Cells

Mast cells have long been considered as effector cells for mediated hypersensitivity and inflammatory responses (27). Its role in the kidney has been largely overlooked in comparison to macrophages and other immune cells. In fact, in a healthy kidney, mast cells release cytokines that protect the kidney from immune damage. They also produce chymases, which produce angiotensin II (28).

Studies have shown that chymase expression is proportional to the degree of renal interstitial fibrosis (29, 30). Mast cell infiltration and increased chymase expression are seen in both glomerulonephritis and ischemia-reperfusion-induced renal fibrosis (31, 32). Mast cells can also promote the proliferation of fibroblasts through intercellular interactions (33). But some researchers have come up with evidence to the contrary. They used mast cell deficient rats to induce nephritis with Puromycin aminonucleoside (PAN) (34). After 6 weeks, it was found that the fibrosis degree of the deficient rats was more serious than that of the wild-type rats, and the expression of TGF-β was significantly higher than that of the wild-type rats. *In vitro* experiments showed that heparin, as an important component of mast cells, could inhibit the expression of TGF-β in rat fibroblasts, suggesting that mast cells may reduce the degree of fibrosis through TGF-β -dependent pathways and play a certain protective role in the kidney.

#### Dendritic Cells

Dendritic cells (DCs) can be divided into plasmacytoid dendritic cells (pDC) and conventional dendritic cells (cDC) (35). Immature DCs have strong migration ability, mature DCs can effectively activate primary T cells, and are in the central link of initiating, regulating, and maintaining immune response (36). It is rarely present in a healthy kidney and can efficiently absorb, process, and present antigens to maintain the stability of the renal internal environment.

Studies have shown that DCs induce and maintain immune responses through migration and maturation in the kidney (37). DCs, although rarely present in normal kidneys, are significantly increased in chronic kidney disease (CKD) and diabetic nephropathy (DN) (38). A 5/6 nephrectomy model was used to induce renal fibrosis, CD1a^+^CD80^+^DCs was found to accumulate in the renal interstitial from 1 week after modeling and peaked at 12 weeks (39). In another study, galectin 3 protects cisplatin-induced acute kidney injury by promoting TLR-2 dependent activation of the IDO1/Kynurenine acid pathway in renal DCs (40). These two studies suggest that DCs were associated with the severity of interstitial fibrosis.

#### T Lymphocyte

T cells are characterized by the expression of co-receptor molecules CD4 and CD8 on their cell surface (41). CD4^+^T cells, also known as T helper cells (Th), recognize antigen/MHC-II complexes on antigen-presenting cells (42) and coordinate the activation of other immune cells, including B cells, macrophages. CD8^+^ cells, on the other hand, called T cytotoxic cells, recognize antigens/MHC-I complexes and are responsible for killing pathogen-infected cells (43). In healthy kidney tissue, T lymphocytes protect the kidney by performing a variety of biological functions to fight infection.

Th cells are thought to play an important role in kidney disease. Th cells were divided into Th1, Th2, Th17 and Treg cells according to the different cytokines secreted (44). Th1 cells mainly secreted IFN-γ, IL-2, IL-12, and so on. Th2 cells mainly secrete IL-4, IL-5, IL-13, etc (45). Th17 cells mainly secrete IL-17. Treg cells mainly secrete IL-10 and TGF-β (46). In a study of rats with idiopathic nephrotic syndrome, proteinuria and focal segmental glomerular injury were observed at 10 weeks of age. Renal T cell infiltration was detected before proteinuria, Th1 and Th2 cells were increased, and Th2 cells were dominant (46). But exactly what the Th1/Th2 equilibrium theory means is still up in the air. The main problem is that the activity of cytokines and other immune messengers rarely falls into strict Th1 or Th2 patterns, and some immune cells, such as Treg cells, stimulate the Th1/Th2 immune system (47). The imbalance of the Th17/Treg ratio plays a role in tissue inflammation, autoimmune, and various diseases. Recently, researchers have proved that the increased ratio of Th17/Treg cells is related to the progression of CKD (48).

Most CD8^+^T cells are cytotoxic, which can induce apoptosis through perforin or Fas/FasL pathway, and can also directly stimulate fibroblast proliferation and extracellular matrix production by secreting TGF-β, IL-4, TNF-a, and other factors, thus aggravating kidney injury (49). Depletion of CD8^+^T cells with antibodies can reduce interstitial dilatation, reduce fibrosis, and alleviate renal parenchymal lesions and renal damage (50). On the contrary, the depletion of CD4^+^T cells aggravated kidney injury, partly because the decrease of CD4^+^T cells caused the increase of CD8^+^T cells (50).

#### B Lymphocyte

B cells have a variety of functions. In addition to the function of antibody secretion, B cells also have the function of releasing inflammatory cytokines, chemokines, and antigen presentation (51). In healthy kidney tissue, B cells make up a small proportion and, together with other immune cells, maintain the stability of the immune microenvironment of the kidney.

There is growing evidence that B cells play an important role in kidney disease. In lupus nephritis(LN), the researchers treated NZB/W lupus mice with a selective histone deacetylase 6 (HDAC6) inhibitor for 4 weeks and showed that HDAC6 inhibition decreased B-cell activating signaling pathways, resulting in a significant reduction in LN symptoms (52). In a clinical trial on patients with IgA nephropathy, the investigators found that toll-like receptor 7 (TLR7) can activate B cells through the TLR7- GALNT2 axis, which produces high levels of galactose-deficient IgA1 (Gd-IgA1) (53).

#### Renal Tubular Epithelial Cells

Renal tubule epithelial cells(RTECs) are composed of a single layer of epithelium and have different morphological characteristics and functions according to the position of renal tubules. For example, in the proximal convoluted tubules, the wall is composed of a single layer of cuboidal epithelial cells (54). The lumen is small and irregular and is an important part of tubular reabsorption. The free surface of the cell has a bristle margin, which enlarges the cell surface area and facilitates reabsorption.

RTECs are involved in the occurrence of kidney injury in many aspects. RTECs can be activated by a variety of cytokines, such as IL-1 and TNF-a produced by monocytes (55). IL-17 is a pro-inflammatory cytokine released by activated T cells. *In vitro*, activation of RTECs with IL-17 can promote the production of IL-6, IL-8, and MCP-1 (56). RTECs are not only important sources of cytokines and chemokines but also can produce pro-fibrotic factors, such as TGF-β, PDGF, CTGF, etc (57). In addition, RTECs are important antigen-presenting cells that can interact with T cells and monocytes.

RTECs are also involved in an important process in renal fibrosis called epithelial-mesenchymal transition (EMT) (58). After EMT, morphological and proteomic changes occurred in RTECs. The so-called EMT is the process in which epithelial cells lose their cellular characteristics, such as polarity and intercellular adhesion, gain the ability to migrate and invade, enter the stroma to obtain new phenotypes, and eventually become mesenchymal cells. In renal fibrosis, EMT refers to the transformation of epithelial cells into myofibroblasts, which are the primary source of the extracellular matrix (59). The expression of various proteins, such as TGF-β, MMPs, FSP-1, and vimentin, increased after EMT. In contrast, some proteins, such as e-cadherin and keratin -18, which are the signature proteins of epithelial cells, are also reduced in expression.

#### Glomerular Mesangial Cells

In healthy renal tissue, glomerular mesangial cells (MCs) only perform the functions of contraction, phagocytosis, and maintenance of normal matrix metabolism. Under pathological conditions, MCs can be transformed from a normal quiescent phenotype to an active proliferation/secretion phenotype with increased extracellular matrix secretion (60). The activation phenotype of MCs has myofibroblast-like characteristics and is characterized by the expression of A-SMA and ED-A fibronectin. After activation, MCs can release a variety of growth factors, such as TGF-β, CTGF, PDGF, etc. through the autocrine or paracrine form to promote self-proliferation (61). At the same time, MCs can synthesize a large amount of extracellular matrix, and mesangial matrix aggregation is the main pathological feature of glomerulosclerosis (62).

In NZB/WF1 mice, the binding of autoantibodies to MCs leads to the initiation of an inflammatory response, an early-stage marker of glomerulonephritis (63). In an *in vitro* model of lupus nephritis (LN), MCs participate in the inflammatory environment of LN by producing cytokines involved in leukocyte recruitment, activation, and maturation. Treatment of MCs with cytokines or patient serum induces TGF-β1 secretion, suggesting that MCs are also involved in the fibrosis process of LN (64).

### Major Metabolites in the Kidney

#### Urea

Urea is a protein metabolite that is produced in the liver and travels through the blood to the kidneys. Some urea is retained in the blood by glomerular filtration and has the opportunity to be transported to the digestive tract as a nitrogen source for microorganisms, while some urea forms tubule fluid and is reabsorbed by the collecting tube of the kidney and returned to the blood.

Urea transporter is a membrane protein that mediates urea transmembrane transport along a concentration gradient, mainly including urea transporter B(UT-B) and urea transporter A(UT-A) (65). UT-A1 is generally distributed in the apical membrane of collecting duct cells in the renal medullary loop (66), UT-A2 is distributed in the descending branch of the loop of the spinal cord (67), UT-A3 is distributed in collecting duct cells in the renal medullary loop basolateral (68), and UT-B1 is mainly distributed in the descending branches of straight small vessels of the nephron (69, 70).

In mouse kidneys, after the deletion of UT-A1 and UT-A3 genes, urine nitrogen excretion increased significantly. After the deletion of UT-B genes, urea in ascending branches of straight small vessels could not penetrate to descending branches of straight small vessels, and the concentration of urea in inner myelin decreased, leading to a decrease in urea circulation in the kidney (71). In conclusion, UT-B, UT-A1, and UT-A3 play irreplaceable roles in the renal urea cycle.

Aquaporin (AQP) is a membrane protein that regulates the infiltration of water into and out of cells (72). It is embedded in the cell membrane and controls the entry and exit of water molecules. Its mechanism of action is similar to urea transporter. So far, 13 aquaporin subtypes, namely AQP1-AQP12, have been identified in animals, but only AQP3, AQP7, AQP9, and AQP10 have clear permeability to urea, which are collectively referred to as water-glycerin channel (AQGP) protein (73–75). Studies have shown that AQGP can also mediate urea transport (76, 77).

In addition to excreting nitrogen, urea also mediates urine concentration through specific urea transport proteins (78, 79). The establishment of the renal medullary osmotic gradient is a necessary condition for the formation of concentrated urine. The active reabsorption of NaCl in the crude segment of the ascending ramus of the medullary loop is the main driving force for the establishment of the medullary osmotic gradient. Urea and NaCl are the main solutes for the establishment of a medullary osmotic gradient.

Proximal tubules are moderately permeable to urea and can reabsorb up to 50% of filtered urea. The collecting tubes in the distal convoluted tubules, cortex, and outer medullary part of the ascending branch of the loop are almost opaque to urea. As the tubule fluid flows through these areas, the water is reabsorbed by collecting tubes in the cortex and the outer medulla, and the concentration of urea in the tubule fluid increases. The collecting tube in the inner medullary region contains UT-A1 and UT-A3, which are activated by several factors and promote the diffusion of urea into the interstitial fluid in the inner medullary region. Urea can re-enter the medullary loop and be reused with a high concentration in the inner medullary region. Urea in the interstitial fluid of the inner medullary is in equilibrium with urea in the collecting tube so that other substances in the interstitial fluid (such as NaCl) are in equilibrium with other substances in the urine to facilitate urine concentration.

Urea transporters can be mediated by several factors in the renal microenvironment that increases urea transport. In short term rapid regulation, Vasopressin signals through two cAMP-dependent pathways: protein kinase A and cAMP-activated exchange proteins (80), high osmotic pressure signals through increased protein kinase Ca, and intracellular calcium (81), thereby increasing UT-A1 and UT-A3 phosphorylation and urea transport (82–84). Vasopressin increases the abundance of UT-A1 and UT-A3 proteins in long-term regulation (85). In addition, urea transporters are affected by low-protein diets (86, 87), adrenal steroids (86, 88), hypokalemia (86), and acidosis (87).

#### Ammonia

Renal ammonia metabolism plays an important role in the maintenance of acid-base homeostasis (88). Almost all urinary ammonia is produced in the kidney, and glutamine in the blood flows through the kidney and is broken down into ammonia in the tubular epithelial cells (89). Urinary ammonia is mainly produced by the decomposition of glutamine, and a small amount comes from the catabolism of other amino acids (90).

In proximal tubules, glutamine uptake requires complete metabolism of glutamine through the involvement of root tip Na+ dependent neutral amino acid transporter-1 and basolateral sodium-coupled neutral amino acid transporter-3 (SNAT3) to produce two NH4+ and two HCO3- ions per glutamine (91). The resulting bicarbonate then passes through the basolateral membrane into the blood vessels *via* the electric-sodium coupled bicarbonate cotransporter isoform 1A (NBCE-1A).

Ammonia reabsorption occurs in the ascending part of the medullary loop. Ammonia is reabsorbed as NH4+ mainly through the transporter NKCC2 and then transported by NHE4, a sodium-hydrogen exchanger on the basolateral membrane (92). NH4+ is a weak acid, and intracellular acidification inhibits ammonia reabsorption (93). Sodium bicarbonate enters cells through electrically neutral sodium-sodium bicarbonate cotransporter subtype 1 (NBCn1) on the basolateral membrane, which appears to buffer intracellular acidification and promote ammonia reabsorption (94).

The collecting tube secretes large amounts of ammonia. The secretion of NH3 is accompanied by the secretion of H+ (95). NH3 secretion seems to be related to the transport of ammonia-specific transporters Rhbg and Rhcg expressed on the collector tube (96, 97). In addition, Na+-K+-ATPase proteins are present on the basolateral side of collecting duct cells, which are involved in ammonia secretion of the intramedullary collecting duct through their ability to transport NH4+ (98).

In summary, ammonia in renal tubular epithelial cells has two pathways: on the one hand, it is discharged into the tubular fluid and excreted in urine; the other is reabsorbed into the blood. NH3 easily passes through the biofilm, while NH4+ does not, so the path of ammonia in the kidney depends on the relative PH of blood and tubular fluid. The PH of blood is generally constant, and therefore actually depends on the PH of the tubule fluid. When the PH value of the tubule fluid is acidic, the NH3 discharged into the tubule fluid combines with H+ to form NH4+ and is discharged with urine. If the PH value of the tubule is high, NH3 is easily reabsorbed into the blood.

Metabolic acidosis can affect ammonia metabolism. During metabolic acidosis, acidosis stimulates the degradation of skeletal muscle protein, which binds to intrahepatic glutamine and increases extrarenal glutamine, leading to increased glutamine flow through the kidneys and increased urinary ammonia production (99, 100). The kidneys remove excess acid from the body by increasing ammonia metabolism (101). Notably, glucocorticoids can modulate ammonia excretion induced by acidosis, possibly by stimulating acidosis-induced extrarenal glutamine increase (102, 103).

Hypokalemia also results in altered ammonia metabolism in the kidneys. Metabolic alkalosis of hypokalemia is often associated with increased bicarbonate production (104). In both adults and children, increased ammonia excretion due to hypokalemia can lead to a negative nitrogen balance and impair health (105).

In addition, a protein diet also regulates ammonia excretion. A high protein diet, especially the intake of sulfur-containing amino acids, lowers PH and promotes ammonia excretion (106). Conversely, a low protein diet reduces ammonia excretion (107).

#### H_2_O, Na+

Water is filtered through the glomerulus and reabsorbed by the renal tubules. The glomerular filtration of protopuria was about 170-180L/d, and the final urine was about 1.5L/d. The reabsorption of water by the kidney can be divided into two forms: passive absorption and active absorption. About 90% of tubule fluid is reabsorbed in renal tubules, and proximal convoluted tubules reabsorb glucose, amino acids, electrolytes, and other substances, and reabsorb water by an osmotic pressure gradient, which is the main form of passive water absorption, accounting for about 80%~90% of water reabsorption (108). The rest are absorbed actively in the medullary loops of renal tubules, distal convoluted tubules, and some collecting tubules, which are regulated by ADH.

There are mainly 8 aquaporins in the kidney, which are AQP1, AQP2, AQP3, AQP4, AQP5, AQP6, AQP7, and AQP11. AQP1 is located at the top of proximal renal tubular epithelial cells, basolateral membrane, and descending branch of the medullary loop. AQP2, AQP3, AQP4, AQP5, and AQP6 are located in the collecting duct, AQP7 is distributed in the brush edge of the proximal convoluted tubule, and AQP11 is located in the endoplasmic reticulum of the proximal tubule cells.

ADH binds with AQP2 in the basement membrane of renal collecting duct epithelial cells to promote the generation of cAMP, activate adenosine cyclase in the perimembrane of tubular cells, increase intracellular cAMP, and then activate protein kinase, phosphorylation of protein located at the luminal surface of the plasma membrane of epithelial cells, and thus increase membrane permeability to water.

It is believed that the water metabolism of the kidney is related to the ball-tube balance. The colloid osmotic pressure in peritubular capillaries can regulate the reabsorption of sodium and water in proximal convoluted tubules. When the glomerular filtration rate (GFR) increases, the filtration excretion fraction (GFR/RPF) also increases. Due to the decrease of protein content in the filtrate, the protein concentration in the blood flowing into the capillaries around the renal tubules increases, and the colloid osmotic pressure in the capillaries also increases, thus promoting the reabsorption of sodium and water in proximal convoluted tubules. In addition, the hydrostatic pressure of peritubular capillaries also regulates the reabsorption of sodium and water in proximal convoluted tubules.

On the other hand, when GFR increased, the amount of Na+ passing through the macula densa also increased, thus increasing the secretion of renin and angiotensin formation in parabulbar cells. Increased angiotensin-2 (AT-2) causes constriction of the entering arterioles, which results in a decrease in GFR and restores the ball-tube balance. Conversely, when GFR decreases, AT-2 production decreases, which causes dilation of the entering arterioles and increase of GFR, and restores the bulbal-tubular balance.

#### Glucose

Renal regulation of glucose metabolism mainly includes gluconeogenesis, glomerular glucose filtration, and proximal convoluted tubules glucose reabsorption. In the fasting state of normal individuals, the kidney produces 15-55g/d glucose through gluconeogenesis, accounting for about 20%-25% of all endogenous glucose. Renal gluconeogenesis is further increased after eating. Renal gluconeogenesis occurs mainly in the proximal convoluted tubules of the renal cortex and is regulated by insulin and catecholamines (109).

Under physiological conditions, the glomerular filtration of approximately 180g of glucose per day is followed by almost complete reabsorption in the proximal convoluted tubules, so urine glucose monitoring should be negative. However, when plasma glucose concentrations reach nearly 10.0mmol/L, the renal glucose threshold will be exceeded, resulting in detectable glucose in urine (110).

The renal glucose reabsorption process is completed in the proximal convoluted tubule S1-S3 segment. The Na+ is pumped out of the cells and into the interstitial fluid by the Na+-k–ATP pump located in the basolateral membrane of renal tubules, thereby reducing the concentration of sodium ion in the cells and forming an electrochemical gradient of about -70 mV. Glucose in the tubule fluid is actively transported to renal tubule cells by the sodium glucose cotransporter (SGLT) in a secondary, inverse concentration gradient. Finally, the glucose transporter (GLUT) binds glucose and changes its conformation, and glucose is then returned to the blood *via* facilitated diffusion from renal tubule cells (111).

SGLT belongs to the SLC5 gene family. SGLT1 and SGLT2 play a role in glucose reabsorption, and their differences in expression and the driving force of their cotransport function help to minimize glucose loss from urine. SGLT2, with high volume and low affinity, is mainly distributed in the proximal S1 segment of the proximal convoluted tubules of the kidney and combines actively transported sodium ions and glucose into the blood circulation in a ratio of 1:1, playing a major role in the renal glucose reabsorption function. SGLT1 is mainly distributed in the brush edge of intestinal mucosal epithelial cells and plays an important role in intestinal glucose and galactose absorption. SGLT1 is also expressed in the distal S3 segment of the proximal convoluted tubules of the kidney with a higher affinity than SGLT2. Glucose that is not bound by SGLT2 is responsible for SGLT1, and glucose and Na+ are reabsorbed into the blood in a ratio of 1:2 (109).

Glucose transporter 1 (GLUT1) and GLUT2 are mainly related to the process of renal glucose reabsorption in the GLUT family. GLUT2 is expressed in the basolateral membrane of renal tubular cells in the S1 segment and is responsible for releasing glucose reabsorbed by SGLT2 into the blood through facilitated diffusion. GLUT1 is responsible for the release of glucose from small tubules into the blood at the proximal convoluted tubule S3 segment (112).

#### Amino Acids

Amino acid metabolism is closely related to the kidney. On the one hand, the absorption, release, metabolism, and excretion of amino acids by the kidney can effectively regulate the level of amino acids in the circulation system and the transport of amino acids between organs. On the other hand, orderly amino acid metabolism is beneficial to regulate renal hemodynamics and protein synthesis, maintain the integrity of renal function, and environmental acid-base balance in the body. Under the normal physiological state, the kidney mainly absorbs glutamine, citrulline, phenylalanine, S-adenosine homocysteine, and proline from the blood, and participates in the synthesis and release of serine, tyrosine, arginine, cysteine, and a small amount of threonine and lysine.

After the kidney absorbs glutamine from the blood, NH4+ and glutamate are mainly metabolized by phosphate-dependent glutamine enzyme, and only a small amount of them metabolized by γ -glutamine transferase in the distal tubules (113). Under the normal physiological state, about 70% OF NH4+ enters the renal vein, and the rest is discharged through urine (114). When PH in the kidney increases, a high concentration of NH4+ inhibits glutaminase activity and increases NH4+ excretion to maintain acid-base balance in the body.

Citrulline is a nitrogenous product of glutamine metabolism in the intestinal tract. Most citrulline is synthesized in the intestinal tract and absorbed by the kidney (115). After the kidney absorbs a large amount of citrulline from the blood, arginine is synthesized and released into the blood with the participation of arginine succinic acid synthase and succinic acid lyase, which accounts for 10-20% of the total plasma arginine (116). Compared with the normal diet, intestinal arginine absorption is reduced in the low-protein diet, resulting in reduced urea synthesis in the liver. At the same time, because citrulline is not taken up by the liver, more citrulline in the low-protein diet enters the kidney to synthesize arginine to maintain normal physiological function.

Arginine can be degraded to guanidine acetic acid and urea, or oxidized by nitric oxide synthase to citrulline and nitric oxide (NO). About 1% of the daily intake of arginine is used to metabolize NO, which is the main source of NO synthesis (117). NO is a small gas molecule that can regulate endothelial cell function and is of great significance in regulating glomerular hemodynamics, maintaining glomerular filtration rate, local vascular tension, and renal blood flow (118, 119).

Asymmetric dimethylarginine (ADMA), symmetric dimethylarginine (SDMA), and N(G)-monomethyl-L-arginine (NMMA) are generated from arginine residues after methylation and proteolytic reaction. Both ADMA and NMMA can inhibit the activity of arginine synthase. NMMA is the precursor of ADMA and SDMA, and the content of NMMA is small but the inhibition effect is strongest. The kidney also plays an important role in the clearance of ADMA and SDMA. The clearance of ADMA mainly relies on renal conversion into citrulline and dimethylamine, and the remaining small amount of ADMA is excreted through urine, while SDMA is mainly cleared through renal excretion (120).

S-adenosine homocysteine is a by-product of methionine methyl transfer reaction and a precursor of homocysteine synthesis. The arteriovenous difference of S-ADENosine homocysteine was up to 40%, indicating that the kidney is the main excretion site of S-adenosine homocysteine (121).

Tyrosine, as a non-essential amino acid, can be synthesized by phenylalanine 4-hydroxylase catalyzed by phenylalanine as a substrate in the body. The synthesis process of tyrosine was first discovered in the liver, and it can also be synthesized in the renal cortex in the later study (122). Moreover, tyrosine synthesized by the kidney is the main source of maintaining the level of tyrosine in the circulatory system (123). Glycine is taken up by the kidneys to synthesize serine, which only accounts for 5-7% of the total amount of serine in the body. High arginine is mainly derived from lysine in the kidney, which can increase intracellular arginine concentration, promote the effective synthesis of NO and improve the dysfunction of endothelial cells and cardiomyocytes (124).

### Causes of Renal Microcirculation Disorders

Dietary preferences can cause gastrointestinal microbiota imbalance and translocation, resulting in renal microcirculation disorders. For example, a high-salt diet can induce oxidative stress in the kidney, resulting in increased renal perfusion pressure and immune cell infiltration, thus leading to kidney damage (125, 126). Meanwhile, under high-salt conditions, serum and glucocorticoid-regulated kinase 1 (SGK1)-mediated phosphorylation of forkhead box of transcription factors O1 (FOXO1) and forkhead box of transcription factors O3 (FOXO3) may lead to instability of Foxp3, thus reducing the inhibitory function of Treg cells (127) (**Figure 4B**). A high protein diet leads to increased urea production in the body. Excessive urea will lead to uremia toxin production in the kidney (128), thus changing the integrity of the intestinal barrier, resulting in the migration of intestinal flora into the blood, resulting in inflammation and cardiovascular diseases (129, 130) (**Figure 1**).

When the imbalance and displacement of microbial flora, a stressor, appears, it will stimulate the activation of the stress response mechanism related to metabolism, leading to excessive passive and active absorption of nutrients by the human body. Excessive absorption of nutrients cannot be efficiently and timely metabolized by the body, easy to lead to nutrient metabolism disorder. For example, lipid substances (triglyceride, cholesterol, etc.), glucose, amino acids, etc., and the disorder of metabolism of these intermediates lead to the impairment of the morphology and function of Treg cells (131–133), the blockage of renal capillaries, and then the occurrence of renal microcirculation disorders (134).

Disturbances in the microcirculation of renal resident cells (RTECs and MCs) impair the exchange of cells with external substances, leading to metabolic disturbances in renal cells (135). Due to the tiny blood vessels front-end blood-supply artery atherosclerosis in silt, the various cells of the kidney can’t get enough nutrients and energy supply, leading to large proteins, lipids, creatinine, urea, renin, prostaglandins, mineral ions, etc. cannot be effectively out of shipping, and metabolism thus can lead to kidney tissue cell metabolism disorder. For example, renal tissue ischemia and hypoxia activate hypoxia-inducible factor 1α (HIF-1α) and destabilize Foxp3 expression, thus inhibiting Treg cell proliferation (136) (**Figure 4B**).

#### Treg Cells Metabolism

**Figure 3:**
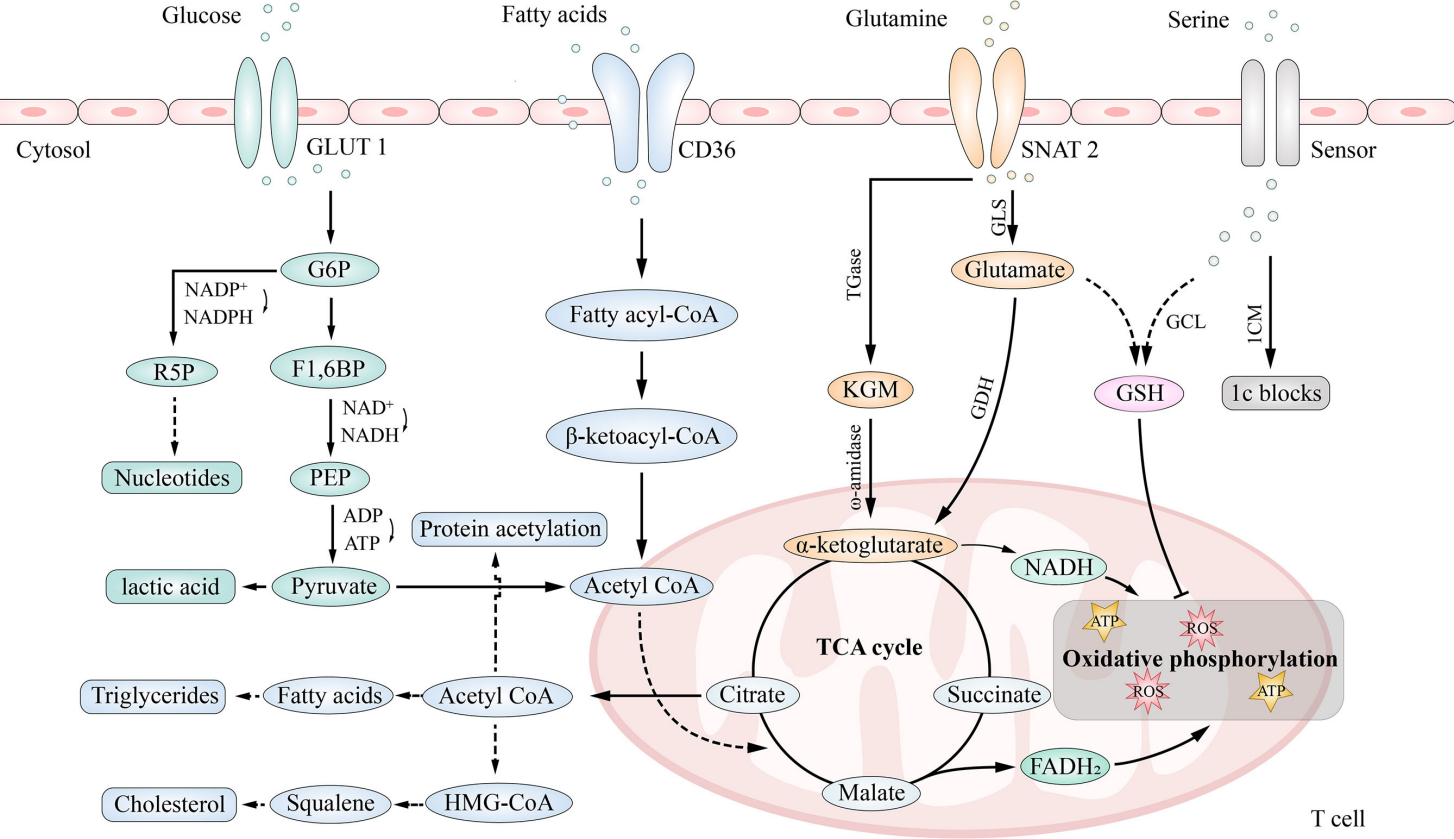
Main metabolic pathways in T cells. T cells generate ATP by glycolysis and oxidative phosphorylation. Pentose phosphate pathway (PPP) is a branch of glycolysis pathway. Cells also generate energy by using glutamine (Gln), which is metabolized by glutaminolysis, and lipids(β-oxidation). Additionally, Serine enters the cell from the extracellular space and then, either enters one-carbon metabolism (1CM), which generating one-carbon (1C) building blocks for anabolism, or produces the ROS scavenger glutathione (GSH).

Cell metabolism is the core of T cell differentiation (137). Resting T cells require little energy production or consumption; however, after activation, their energy demand increases significantly, and they use glucose, amino acids, and fatty acids to meet this requirement (138, 139). An overview of the metabolic pathways is shown in **Figure 3**. Treg cells mainly utilize fatty acid and pyruvate oxidation (mitochondrial oxidative metabolism) to produce energy, which has a different signal and metabolic characteristics from other T cells (139).

#### Glucose Metabolism in Treg Cells

Glucose is required for the activation and proliferation of Treg cells. They can make use of glycolysis and oxidative phosphorylation for energy production (140). Glycolysis occurs in the cytoplasm by converting glucose to pyruvate (producing two ATP molecules), which is converted to lactate by lactate dehydrogenase A (LDH-A), or to acetyl-CoA by pyruvate dehydrogenase (PDH), which then travels to the mitochondria to participate in the tricarboxylic acid (TCA) cycle, producing ATP (36 molecules) through oxidative phosphorylation (141). Moreover, the pentose phosphate pathway (PPP) that branches from the glycolysis pathway converts glucose-6-phosphate to ribose-5-phosphate for the synthesis of nucleotides.

Foxp3 itself inhibits glycolysis and promotes oxidative phosphorylation (OXPHOS), while Foxp3 deficiency dysregulates mammalian target of rapamycin complex 2 (mTORC2) and promotes glycolysis (132, 142). Upregulation of GLUT1 in Treg cells inhibited Foxp3 expression (142, 143). A study showed that Treg cells had higher levels of C2 and C4-OH carnitine, higher expression of fatty acid transport protein carnitine palmitoyltransferase 1A (CPT1A) and electron transport chain component cytochrome C, and lower levels of GLUT1, a key protein expressed in pyruvate, lactic acid, and glycolysis pathways, suggesting that the energy of Treg cells depends more on oxidative phosphorylation than glycolysis (144). Moreover, some studies have shown that a high glycolysis rate is not conducive to the differentiation of Treg cells. Conversely, inhibition of glycolysis can promote the formation of Treg cells (145, 146). It can be explained because, mechanically, glycolysis requires activation of MYC proto-oncogene (MYC) and Foxp3 binds to the promoter of MYC to inhibit expression of MYC and MYC-dependent transcripts (147).

**Figure 4:**
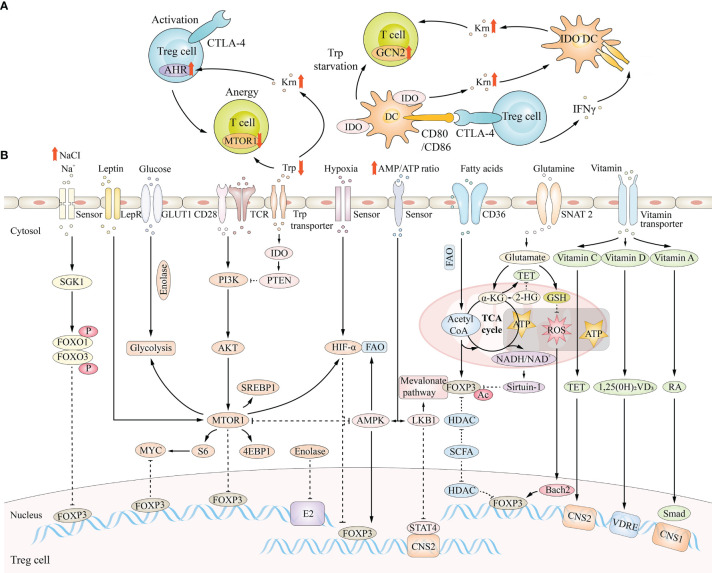
Metabolic regulation in Treg cells. **(A)** Regulatory T cells have a crucial role in establishing an IFN-γ-rich environment that activates Indoleamine 2, 3-dioxygenase (IDO)- and IDO+ dendritic cells (DCs), either by forward signaling to DCs or by direct production of the cytokine. **(B)** Cell-intrinsic metabolic programs and environmental factors that can modulate FOXP3 expression ultimately affect Treg cells fate.

The glycolytic enzyme enolase 1, relocating from the cytoplasm (where it regulates the glycolysis pathway) to the nucleus, is required for the induction and function of human pTreg cells following suboptimal T cell receptor (TCR) stimulation of T cells in the periphery (138). In the nucleus, enolase 1 binds to the epigenetic promoter region of the Foxp3 gene to inhibit transcription of specific Foxp3 exon-2 (E2) (148) (**Figure 4B**).

As mentioned above, pyruvate can be converted to lactic acid by LDH-A under anaerobic conditions. For example, in ischemic tissue, due to the accumulation of lactic acid caused by ischemia and hypoxia, lactic acid mediates increased HIF-1α production and inhibits pTreg function, which may lose the metabolic advantage of function under low glucose conditions (149). However, in prostate cancer models, lactic acid produced by cancer-associated fibroblasts (CAFs) stimulates Treg proliferation by promoting Foxp3 activation (150). These two contradictory results are currently unclear and may be interpreted that lactic acid increases the number of Treg cells, and the inhibitory ability of Treg cells decreases during Treg proliferation. In addition, lactic acid can also be converted to pyruvate through lactate dehydrogenase B (LDH-B) (151). One study showed that in tumor cells, oxidation of lactic acid to pyruvate changed the ratio of NAD+/NADH, thereby activating the Silencing information regulator 2 related enzyme 1/proliferator-activated receptor γ coactivator-1α (Sirtuin1/PGC-1α) axis of NAD+ dependent deacetylase, enhancing the mitochondrial metabolism and invasion ability of prostate cancer cells (152).

Moreover, glycolysis of Treg cells can be inhibited by the binding of effector molecules on cytotoxic T lymphocyte antigen 4 (CTLA4) and programmed death 1 (PD-1) found on the surface of Treg cells (153). In turn, the inhibition of glycolysis can also suppress the migration of Treg cells, and to meet their glucose demand, Treg cells upregulate insulin receptors (154). Several recent studies have indicated that Treg mobility is regulated by the metabolism of glucose through glycolysis, *via* glucokinase (GCK) activation and phosphoinositide 3-kinase (PI3K)- protein kinase B (Akt) pathways (155).

#### Fatty Acid Metabolism in Treg Cells

Fatty acids are transformed into acyl-coenzyme A (FA-CoA) in the cytoplasm, and FA-CoA enters mitochondria under the action of carnitine palmityl transferase I (CPT I) and carnitine palmityl transferase II (CPT II) (156). After β-oxidation, acetyl-CoA is formed and enters the tricarboxylic acid cycle. Fatty acid oxidation requires the involvement of four enzymes that produce NADH and xanthine dinucleotide (FADH2), which are used by the electron transport chain to produce ATP (157). Acetyl-CoA in the mitochondria is transported to the cytoplasm through the citrate-pyruvate cycle for the synthesis of fatty acids, triglycerides, cholesterol, and protein acetylation (158). The fatty acid synthesis requires acetyl CoA carboxylase 1 (ACC1) (159), and the cholesterol synthesis requires the participation of acetyl-CoA and hydroxymethylglutaryl-CoA (160).

Acetyl-CoA may promote the acetylation of Foxp3 protein by activating lysine acetyltransferases (KATs) and prevent Foxp3 protein ubiquitination degradation, thus helping to maintain the function of Treg cells (161), while NAD+/NADH activates the deacetylase activity of Sirtuin-1 and inhibits Foxp3 protein (162) (**Figure 4B**). CPT I, a rate-limiting enzyme in fatty acid oxidation (FAO), enhances FAO efficiency, and adenosine monophosphate activated protein kinase (AMPK) induces CPT I expression (163, 164). In fatty acid synthesis (FAS), acetyl-CoA is carboxylated into malonyl-CoA ACC1, the rate-limiting enzyme, and its phosphorylation can inhibit FAS (165). ACC1 inhibits the polarization of Treg cells and inhibition of ACC1 can promote the induction of Treg cells *in vivo* and *in vitro* (166). The inhibition of Fatty acid Binding protein 5 (FABP5) in Treg cells can trigger the release of mitochondrial DNA (mtDNA) and the subsequent cyclic GMP-AMP synthase-stimulator of interferon genes (cGAS-STING) dependent type I interferon (IFN) signal transduction, thus inducing the production of the regulatory cytokine interleukin-10 (IL-10) and promoting the inhibitory activity of Treg cells, suggesting that FABP5 maintains mitochondrial integrity and can regulate the function of Treg cells (167).

TTreg cells and pTreg cells together constitute the Treg cell population *in vivo*, and they have different metabolic characteristics. PTreg cells can utilize both the mevalonate pathway and end products of glycolysis for the synthesis of fatty acids (168). During the induction of Treg cells in the periphery, increased expression of Foxp3 reprograms the cell to exert action in low-glucose environments (169). Therefore, the cell preferentially metabolizes lipids over glucose to maintain the suppressive function of pTreg cells, which maintains peripheral immune tolerance during tissue injury, even under metabolically challenging conditions (e.g., in ischemic tissues) (170). TTreg cells are induced and maintained by exogenous fatty acids (138). For example, Treg cells in adipose tissue express leptin receptors, and the leptin/leptin receptor axis induces an mTOR metabolic state to inhibit Foxp3 expression (171, 172) (**Figure 4B**). In addition, Treg cells in tissues are involved in suppressing inflammation and have regenerative functions in wound healing (173).

Short-chain fatty acids (SCFAs), including acetic, propionic, and butyric acids, are produced by dietary fiber and other undigested carbohydrates in the colon (174). At the molecular level, SCFAs inhibit histone deacetylase (HDAC) in Treg cells in colon tissue and enhance histone acetylation at Foxp3, promoting pTreg cells formation (175). Interestingly, SCFAs-induced IL-10+pTreg cells were not associated with suppression of the immune response in kidney hydronephrosis (C2RD), since the number of Th1 and Th17 cells increased as the number of regulatory T cells increased, suggesting a general increase in SCFAs-induced T-cell response in C2RD (176).

MTOR drives FAS and cholesterol production in Treg cells, while SCFAs can activate the mTOR pathway (177, 178). Studies have shown that the activity of mTOR- S6 kinase (S6K) in SCFAs-induced Treg cells is increased, revealing that SCFAs may regulate Treg cells through the mTOR-S6K pathway (173, 176). Meanwhile, with the activation of the mTOR pathway, SCFAs also enhance the activity of signal transducers and activators of transcription 3 (STAT3) (176). In addition, impaired Treg homeostasis in mTOR deficient mice was associated with defective lipid biosynthesis (177).

OX40 can also trigger the proliferation of lipid-rich Treg cells in naive mice (168). It has been found that OX40/OX40L signaling occurring in the hepatocellular carcinoma microenvironment may be directly involved in the FAS of Treg cells (179).

In addition, researchers found high expression of peroxisome proliferation-activated receptor gamma (PPARγ) in TUM-Treg cells (Treg cells extracted from the tumor bed) (168). PPARγ is a nuclear factor that controls fatty acid uptake and FAS in adipose tissue (180), and it is believed that excessive FAS induces PPARγ expression.

#### Amino Acid Metabolism in Treg Cells

Amino acids also play a crucial role in Treg cell regulation. 2-Hydroxyglutarate (2-HG), the metabolite of glutamine, can lead to hypermethylation of the Foxp3 gene locus and then suppress Foxp3 transcription (181); this action further inhibits Treg generation. Tryptophan can produce kynurenine, which is able to combine with the aryl hydrocarbon receptor and then accelerate pTreg generation (182). Moreover, the enzymes that pTreg express participates in the synthesis of amino acids, which play an essential role in the proliferation and function of Treg cells (183, 184). Indoleamine-2,3-dioxygenase (IDO) is expressed on Treg cells and can inhibit mTOR signaling by phosphatase and tensin homolog (PTEN), thus promoting the generation of Treg cells (185). After tryptophan is metabolized by IDO, the metabolite kynurenine will bind to the transcription factor aryl hydrocarbon receptor (AHR), thereby activating Foxp3+ Treg cells (186), and these Treg cells will reverse or inhibit the activity of effector T cells. Kynurenine could also recruit other cell types to the regulatory response, including dendritic cells (DCs), in which the function of IDO is inhibited posttranslationally (187) (**Figure 4A**).

#### mTOR/AMPK Signal Pathway in Treg Cells

mTOR, a member of the phosphatidylinositol 3-kinase-related kinase family, induces the expression of multiple genes that play a key role in a variety of metabolic processes and is necessary for Treg differentiation, function, and survival (177) The increase in mTOR pathway activity has a negative impact on the generation and function of Treg cells (188) Transient TCR stimulation can induce the PI3K-Akt-mTOR signaling pathway to antagonize the expression of Foxp3 (189).

MTOR consists of the protein complex mTOR complex 1 (mTORC1) and mTOR complex 2 (mTORC2). Increased mTORC1 activity is not only a typical characteristic of Th1 cells and Th17 cells differentiation (190) but also has a negative impact on the generation and function of Treg cells (188). The mTORC1 signaling pathway in Treg cells is inhibited by serine/threonine protein phosphatase 2A (PP2A). In the absence of PP2A, the glycolysis and oxidative phosphorylation rates of Treg cells were increased (191), as well as the expression of small subunit 1 (LAT1), a neutral amino acid transporter dependent on mTORC1 activity (177). The increase of glycolysis, oxidative phosphorylation, and LAT1 expression made Treg cells develop into Teff cells. However, whether PP2A has an effect on mTORC2 requires further study (192).

In fact, the relationship between mTORC1 and mTORC2 remains at odds. Aysegul V. Ergen et al. suggested that rapamycin inhibited mTORC1 but not mTORC2 (193). However, Rosner M et al. suggested that chronic treatment with rapamycin also inhibited mTORC2 activity (194). Takahito Kawata et al. argued that mTORC1 negatively regulates mTORC2 (195); Wang et al. believed that mTORC1 maintained mTORC2 activity, while mTORC2 negatively regulated mTORC1 signal activation (196). These differences may be related to researchers’ experimental conditions and models, which need further study.

IDO activity reduces local tryptophan availability in the vicinity of Treg cells in the tumor microenvironment (197). A low concentration of tryptophan inhibits mTORC2 through protein kinase and prevents its phosphorylation of Akt, which helps maintain the inhibitory function of Treg cells, suggesting that the MTORC2-Akt signaling pathway has a negative regulatory effect on the differentiation of Treg cells (198).

AMPK can be activated by Treg cells and can inhibit mTORC1, reduce the expression of GLUT1 and promote fatty acid oxidation (199). Liver kinase B1 (Lkb1) is considered to be an AMPK-independent metabolic sensor in Treg cells because it stabilizes the expression of Foxp3 by changing the methylation state of CNS2 (200).

#### Other Metabolic Pathways in Treg Cells

As mentioned above, SGK1-mediated phosphorylation of FOXO1 and FOXO3 may lead to instability of Foxp3 under high salt conditions, thus reducing the inhibitory function of Treg cells (127). However, in another study, high salt only inhibited the function of tTreg cells and had little effect on TGF-β -induced iTreg cell function (201). This may be due to the different disease models selected by the authors.

Excessive urea will lead to uremia toxin production in the kidney, studies have shown that uremia patients have reduced Treg cells numbers and impaired function (202, 203) However, the specific mechanism of how urea acts on Treg cells remains unclear.

HIF-1α, which is activated either directly by hypoxia or *via* mTORC1, destabilizes Foxp3 expression (204). Moreover, HIF-1α is downregulated by 2-HG through inhibiting the activity of prolyl hydroxylase (205). The vitamin A metabolite RA increases Foxp3 gene expression by maintaining Smad activation (206). Vitamin C, together with Tet methylcytosine dioxygenase, increases Foxp3 expression (207). The vitamin D3 metabolite 1,25(OH)2VD3 stabilizes Foxp3 gene expression by maintaining the state of the VDRE region (208) (**Figure 4B**).

In addition, we summarized some drugs that target metabolic pathways; for example, 2-deoxy-d-glucose (2-DG), a drug that can compete with glucose in binding to hexokinase II (HKII) to inhibit cellular glycolysis activity and regulate the glycolytic pathway, induces Treg cell differentiation and suppression and alleviates the progression of systemic lupus erythematosus (SLE) in TC mice (209). More drugs and further details are given in **Table 1**. These drugs can change the number and function of Treg cells by targeting their respective metabolic pathways, thus alleviating the progression of diseases.

**Table 1:** Inhibitors of the metabolic pathways, their influence on Treg cells and disease applied.

Related Mechanism Pathways	Drugs	Pharmacological Effects	Influence on Treg Cells	Other biological Functions	Experimental Subject	Associated Disease	Reference
carbohydrate metabolism	CG-5	Decrease Glut1 expression	Increase Treg cells differentiation	*In vitro*: block glycolysis in CD4+ T cells	Lupus-prone mouse model	SLE	(220)
	2-DG	Compete with glucose in binding to HKII to inhibit cellular glycolysis activity and regulate the glycolytic pathway	Induce Treg cells differentiation and suppression	*In vivo*: dampen Th1 and Th17 cells development	Lewis rats	GBS	(221)
				Decreased ECAR and OCR in TC CD4+ T cells	TC mice	SLE	(210)
	DCA	Inhibit the dephosphorylation and deactivation of PDC to keep PDC active	Increase Treg cells expansion	Inhibit Th17 cells survival and proliferation	C57BL/6J mice	EAE	(222, 223)
	Metho- trexate	Act by competitive inhibition of dihydrofolate reductase to deplete One-carbon metabolism	Increase Treg cells expansion	Deplete purine biosynthesis enzymes	Patients with RA and healthy controls	RA	(224)
Lipid metabolism	Piogli- tazone	Activate PPARγ and high affinity binding to the PPARγ ligand-binding	Induce VAT Treg cells	Decrease the elevated serum levels of both creatinine and CK-MB	C57Bl/6 mice	Obesity	(225, 226)
	Sora-phen A	Lower cellular malonyl CoA, attenuate DNL and the formation of fatty acid elongation products derived from exogenous fatty acids	Induce Treg cells differentiation	*In vivo*: inhibit TH17 cell–associated inflammatory diseases	TACC1 mice	EAE	(211, 227)
	TOFA	Inhibit ACCA to decrease fatty acid synthesis and induce caspase activation	Inhibit Treg cells proliferation	*In vitro*: reduce the MCA38 cell viability in a dose-dependent fashion	Tumor-bearing mice	Tumor	(171, 228)
	Etomo- xir	Bind irreversibly to the catalytic site of CPT-1 to inhibit CPT-1 and up-regulate fatty acid oxidase activity	Abrogate Treg cells development and suppressive function	Reduce the production of pro-inflammatory cytokines in MOG specific T cells and promote their apoptosis	C57BL/6J mice	MS	(229, 230)
Amino acid metabolism	DON	Inhibit glutaminase and glutamine transporters	Promote Treg cells generation and frequency	Decrease IFN-γ production and proliferation in activated CD4+ and CD8+ T cells	C57BL/6 mice	Skin and heart transplantation	(231, 232)
mTOR/AMPK signal pathway	Rapa-mycin	Block mTOR downstream targets, such as p70S6K phosphorylation and activation	Enhance nTreg cells proliferation and function	Suppress proliferation of CD4+ CD25- effector T-cells	Patients with type 1 diabetes and healthy controls	Type 1 diabetes	(233–235)
	Metfor-min	Activate AMPK in liver cells leads to decreased ACC activity, induction of fatty acid oxidation, and inhibition of adipogenic enzyme expression	Induce Treg cells differentiation	Inhibit IL-17, p-STAT3, and p-mTOR expression	C57BL/6 mice	IBD	(236, 237)

2-DG, 2-deoxy-d-glucose; ACC, acetyl-coa carboxylase; CK-MB, creatine kinase-mb; DCA, dichloroacetate; DON, 6-diazo-5-oxo-L-norleucine; EAE, experimental autoimmune encephalomyelitis; ECAR, extracellular acidification rate; GBS, Guillain-Barré syndrome; IBD, inflammatory bowel disease; MS, multiple sclerosis; OCR, oxygen consumption rate; RA, rheumatoid arthritis; SLE, systemic lupus erythematosus; TOFA, 5-tetradecyl-oxy-2-furoic acid.

#### The Role of Treg Cells in Acute Kidney Injury

Acute kidney injury (AKI) refers to a clinical syndrome in which renal function declines rapidly in a short period caused by a sudden or continuous decline in the glomerular filtration rate (212, 213). AKI has multiple etiologies, with hypovolemia, ischemia-reperfusion injury (IRI), exposure to nephrotoxic agents, and sepsis among the major causes. The immune response mediates the various stages of the occurrence, development, and repair of AKI, and Treg cells play a significant role in the entire developmental stage of AKI (210, 214). Although there is no clinical study of Treg cells in AKI, they have been indicated to protect and repair the kidney after AKI in animal models (215).

Abnormal metabolism in AKI affects the signaling pathways and the extracellular matrix environment, thereby affecting the differentiation of Treg cells. In AKI patients, due to mitochondrial damage and impaired FAO metabolism, as well as decreasing peroxisome proliferation-activated receptor alpha (PPARα) activity and decreasing peroxisome PGC-1 expression, the accumulation of triglycerides in AKI patients result in obvious lipid metabolism abnormalities (216–219). Treg cells express G protein-coupled receptor 43 (GPR43) in mice, which when bound to SCFAs results in enhanced differentiation and function (238). Recent work by Field C. et al. demonstrated that inhibiting the lcFA-FAO metabolic pathway may be more favorable as an approach to increasing the suppressive function of Treg cells (167). It is plausible that various intermediates produced during FAO, such as acetyl-CoA, and reduced FADH/NADH, could interfere with Treg cell function through yet unknown mechanisms (167). Mitochondrial dysfunction is also one of the important characteristics of AKI (239); the accumulation of cytochrome C in the mitochondria causes the oxidative respiratory chain to fail to proceed normally, and mitochondrial respiration is weakened, which further affects the metabolism of lactic acid in the kidney tissue, causing metabolic acidosis (240). In this situation, Treg cells can convert lactate to pyruvate. Moreover, Foxp3 can modulate LDH to prevent lactate formation and form pyruvate (241). While lactate may negatively impact T-cell proliferation as a whole, it does not impact Treg cell immunosuppression (242). There are also serious changes in protein metabolism in AKI, and the rapid catabolism of proteins leads to a negative nitrogen balance (243). In a long-term high catabolic state, the activity and metabolism of Treg cells will be affected, resulting in a weakened immune response and anti-infection mechanism (244). Active metabolism in AKI will cause hypoxia, which is associated with increased levels of the HIF-1 complex (245). HIF-1α forms a complex structure with its counterpart HIF-1β, which then binds to specific hypoxic response elements (HREs) (243) to influence Treg cell metabolism/function.

#### Ischemia–Reperfusion Injury (IRI)

The phenomenon that after blood reperfusion is resumed under certain conditions, some animals or patients have cellular functional metabolic disorders and structural damage that are not reduced but aggravated this is called IRI (246). IRI is a vital cause of AKI and a serious complication secondary to major surgery (247). Endogenous Treg cells can mediate immune responses, reduce the existence of costimulatory molecules after renal IRI, and improve renal IRI (248). Oxidative stress, inflammation, and apoptosis are well-known characteristics of the kidney after AKI (249). *In vitro* and in mice, IRI causes persistent mitochondrial damage and energy loss (250), increased reactive oxygen species (ROS) generation, and decreased ATP synthesis in iTreg/pTreg cells. Upregulation of Treg cell lipogenic genes in the kidneys of IRI mice leads to an associated elevation of lipid deposition (250, 251), indicating the presence of excessive FAS in Treg cells of IRI mice. The tryptophan metabolite kynurenine was increased in plasma and kidney tissues from IRI mice (250, 251). After the elimination of pTreg/iTreg cells in renal IRI mice by anti-CD25 + antibody *in vivo* and *in vitro*, renal injury and inflammation were aggravated, and renal function and mortality continued to deteriorate (252). Adoptive transfer therapy of iTreg cells after IRI can increase the repair rate of mouse kidneys (253). In addition (254), Treg effectively prevents the accumulation of neutrophils and mononuclear phagocytes during renal reperfusion, and its pathway has not been fully understood yet.

#### Sepsis-Induced Acute Kidney Injury

Severe sepsis can also lead to AKI (255). Although the pathophysiological mechanisms are not fully understood, it is clear that the inflammatory cascade characteristic of sepsis is associated with AKI (256). Different from AKI caused by IR, renal tubular cells in septic AKI are slightly vacuolated and a large number of renal tubular cells undergo apoptosis, without obvious renal tissue necrosis (257). In the septic AKI mouse model, renal tubular cell apoptosis was reduced and renal function was significantly improved after Treg cells were removed, which was completely contrary to the findings in IR mice, since the depletion of Treg cells led to the deterioration of renal function after IR (254). There was no significant change in renal function after IL-10 blockade in IR mice, but in septic AKI, renal function was significantly improved after IL-10 blockade, suggesting that IL-10 reduced the proliferation of Treg cells, thereby improving the survival rate of patients with sepsis (258). The opposite role of Treg cells in septic-induced AKI and IR-induced AKI maybe that Treg cells only play a protective role in aseptic inflammation-mediated AKI, which needs further study to explain.

#### Cisplatin Nephrotoxicity

Cisplatin, one of the most effective chemotherapeutic drugs, can induce damage in the renal vasculature, which leads to reduced blood flow and ischemic injury in the kidney, thereby inducing an AKI model (259). Cisplatin has been widely used to treat malignant tumors of various organs. It is known that cisplatin concentrates on epithelial cells in the proximal tubule S3 segment (260), where it induces necrotic and apoptotic cell death and is associated with an extensive pro-inflammatory immune response. Salt may ameliorate symptoms (261, 262). Some studies have shown nephrotoxicity in clinical trials of cisplatin chemotherapy (259), and the use of cisplatin is limited because of its nephrotoxicity (263). CD4+CD25+Foxp3+ Treg cells showed a protective effect in the cisplatin nephrotoxicity test in mice (264). Oxidative stress has been considered an essential component that results in cisplatin nephrotoxicity both *in vivo* and *in vitro (* 265, 266). Cisplatin aggregates in the mitochondria of renal epithelial cells and disrupts the respiratory chain, resulting in a decrease in ATP production and an increase in ROS production, which cause inhibition of mitochondrial activation (259, 263, 264). Mitochondrial dysfunction and oxidative stress exist in cisplatin-mediated acute renal injury (267), which causes impaired synthesis of Treg cells (268).

With the in-depth understanding of AKI disease and Treg cells, AKI, which was previously thought to have little relationship with immune abnormalities, is partly caused by abnormal metabolism of immune cells, such as Treg cells. However, the immune cells involved in AKI disease are not only Treg cells. What is the proportion of Treg cells interacting with other immune cells in AKI disease? Does targeted Treg therapy for AKI affect other abnormalities in immune cell function? How do Treg cells affect intrinsic renal immune response in cisplatin-induced AKI? These all require follow-up research.

#### The Role of Treg Cells Metabolism in CKD

CKD is the chronic process resulting from a variety of kidney diseases, as well as a heterogeneous illness influencing the morphology and function of the kidney (269). A diverse set of components can activate various molecular mechanisms of kidney damage, such as genes, metabolism, autoimmunity, malignancy, toxins, and the environment (270). All these injuries contribute to different categories of vascular, glomerular, and tubulointerstitial renal diseases, which culminate in decreased kidney function and result in CKD syndrome (271).

**Figure 5:**
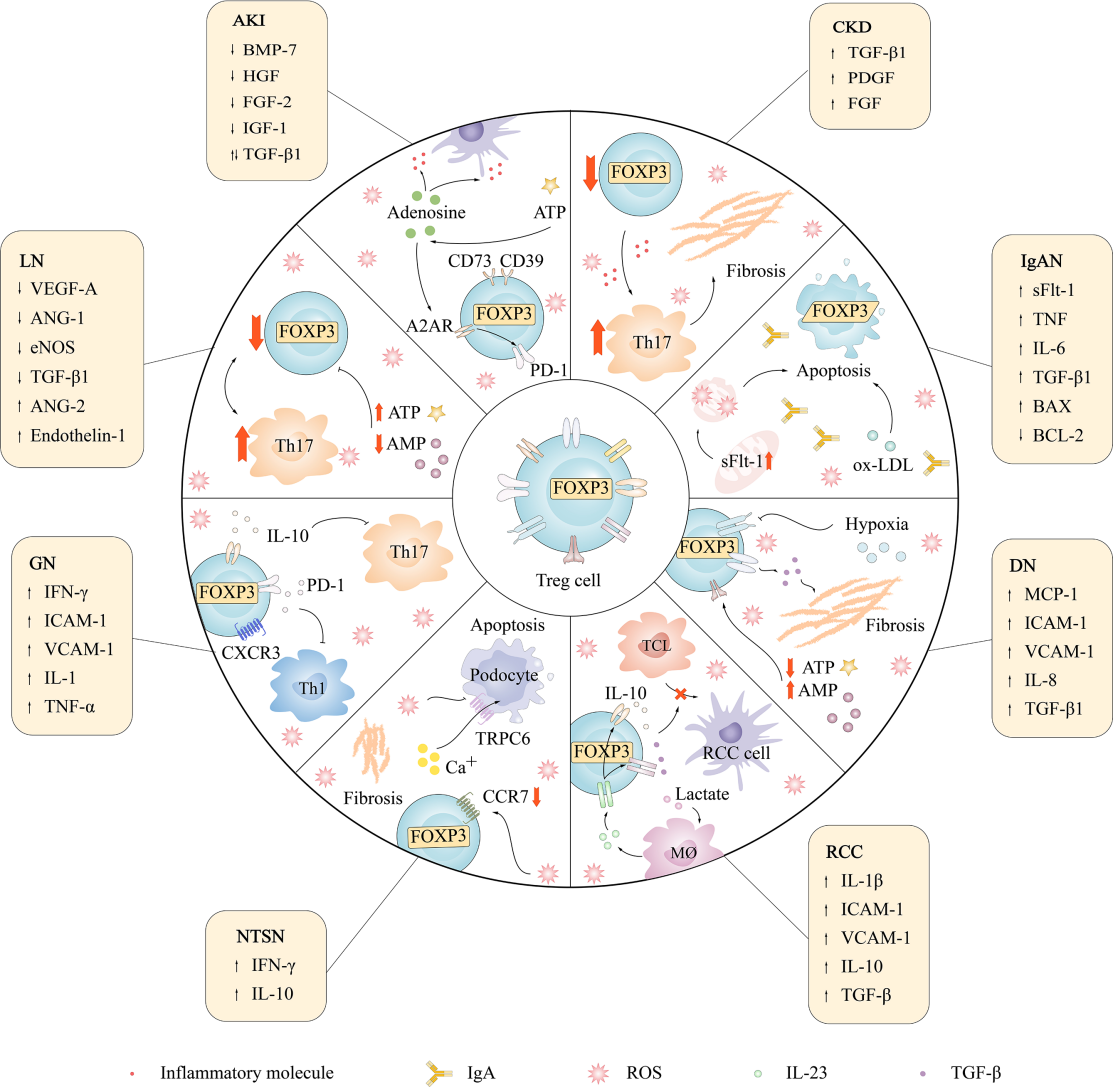
The relationship between Treg cells and renal diseases and changes of cytokines.

Tissue damage in CKD is directly or indirectly mediated by the immune system, and the dysfunction of immune cells promotes CKD inflammation and renal fibrosis (272). Treg cells play a protective role in CKD by inhibiting inflammation and immunity, but the number of pTreg cells in the peripheral blood of CKD patients is significantly reduced (273). TGF-β1 is an inducer of Treg cells, which are released after renal cell injury (274). Treg cells can be transformed into Foxp3+IL-17+ T cells under inflammatory conditions in the kidney and then produce a large amount of TGF-β1, leading to CKD inflammation and renal fibrosis (275) (**Figure 5**). A study illustrated that an elevated ratio of Th17 cells and a reduced ratio of Treg cells exist in CKD patients, reflecting that an enhanced Th17/Treg cell rate is related to the progression of CKD and the severity of kidney disease (48).

The state of reduced renal function that results from CKD causes marked alterations in Treg cell metabolism. Typical alterations include increased intracellular ROS (276), high levels of 8-hydroxy-2 deoxyguanosine (8-OHdG) (277), enhanced carbohydrate metabolism (278), and abnormal lipid metabolism (279). ROS stabilize the nuclear factor of an activated T cell (NFAT) in the nucleus and bind to CNS2 to promote Foxp3 expression (280), and interestingly, it can directly inhibit the enzymatic activity of several elements in the cellular respiratory chain, while complex III per se is key to promoting Treg cell suppressive function (242, 281). 8-OHdG (277) is a marker of oxidative DNA stress. Oxidative stress can induce the activation of the PI3K/Akt/mTOR signaling pathway and induce the phosphorylation of mTOR in CKD patients (282), increasing mTOR activation in cells, which negatively affects the protective effect of Treg cells on the kidney. Dietary fiber, a kind of carbohydrate, can be converted into SCFAs, which are a main source of nutrients for Treg cells. Therefore, a high fiber diet can potentially attenuate systemic inflammation and CKD progression (278). Dyslipidemia in CKD patients is largely due to changes in low-density liptein cholesterol (LDL-C) levels (283). Researchers have indicated that proteasome inhibition by ox-LDL leads to CD4+CD25+ Treg apoptosis, affecting the number and suppressive capability of these Treg cells in chronic hemodialysis (HD) patients (284). In addition, in Ldlr−/− mice, Treg cells were found to control very-low-density lipoprotein (VLDL) levels by regulating the lipoprotein binding protein, sortilin 1, protecting against the development of CKD (279).

#### Diabetic Nephropathy

Diabetic glomerulosclerosis is a leading factor of CKD and end-stage renal disease (ESRD), and an autoimmune renal disease secondary to diabetes mellitus type 1 (T1DM) or diabetes mellitus type 2 (T2DM) (285). Diabetic nephropathy (DN) is characterized by glomerular hypertrophy, basement membrane thickening, the accumulation of extracellular matrix components, and kidney inflammation, which are crucial in promoting the development and progression of DN (286). Recently, the morbidity and mortality of DN have been increasing rapidly worldwide (287–290). Some studies have demonstrated that there is an imbalance in the Treg/Th17 cell ratio in patients with T1DM, which may be related to the progression of microangiopathy (291). Treg cells are correlated with diabetes and DN, and T2DM patients have a low level of Treg cells relative to Th1 or Th17 (292, 293). The increase in the number of Th17 cells leads to an increase in the release of pro-inflammatory factors such as IL-17, which triggers local tissue inflammation and promotes the development of DM complications (291). The level of Treg cells in the peripheral blood of patients with type 2 diabetic nephropathy (T2DN) decreased, and the use of anti-CD25 antibodies to eliminate Treg cells aggravated kidney damage, while adoptive transfer of Treg cells reduced blood sugar and improved diabetic nephropathy (210).

The progression of diabetic nephropathy is also influenced by oxidative stress (294), lipid metabolism (295), and mTOR activation (296). Excessive ROS can damage mitochondria and increase the production of lipid peroxides (297) in Treg cells. Normally, sodium-dependent glucose transporter 2 (SGLT-2)-reabsorbed glucose is utilized by mitochondria to synthesize ATP by oxidative phosphorylation (OXPHOS) (298). However, mitochondrial dysfunction occurs following inhibition of OXPHOS, which results in decreased ATP production (299), and loss of mitochondrial membrane potential (ΔΨm) and can ultimately lead to increased ROS from various sites of the electron transport chain (ETC), including complex III, which is key to promoting Treg cell suppressive function (298). Interestingly, Treg-specific knockout of complex III is associated with reduced immunosuppressive capacity and increased DNA methylation status, but it has no relevance to FOXP3 expression (281).

Abnormal metabolism and accumulation of lipids in the kidney play a crucial role in the pathogenesis of DN (300). Abnormalities in lipid metabolism make Treg cells unable to obtain sufficient energy to complete their functions (301). During a state of high ATP consumption, there is a proportional increase in intracellular AMP and HIF-1α (302) (**Figure 5**). The proportional increase in AMP leads to AMPK phosphorylation and activation by liver kinase B1 (Lkb1) (300), which is crucial for Treg cell metabolism and function. Excessive HIF-1α leads to decreased Treg differentiation, as HIF-1α can promote FOXP3 ubiquitination and subsequent proteasome degradation (136, 146)

Activation of the mTOR pathway is upregulated in renal diseases such as DN (303). The PDK1/Akt/mTORC1 signaling pathway is activated in the glomerular mesangial cells of patients with DN, which induces the high expression of S6K1 and 4EBP1 (304), causing excessive cell proliferation and hypertrophy (305). mTORC1 activity plays an important role in Treg cell activation, function, and increased metabolic demands. mTORC2 is also involved in regulating hypertrophy of mesangial cells induced by high glucose, and inhibition of mTORC2 can reduce the phosphorylation levels of PKC II and Akt, suppress mTORC1 activity, and prevent mesangial cell hypertrophy (306). Some data suggest that mTORC2 inhibition promotes Treg cell activation status, Th2-like differentiation, and immunosuppressive function (142). In addition, the PI3K/AKT/mTORC1 signaling pathway is involved in extracellular matrix (ECM) deposition and tubulointerstitial fibrosis. On the one hand, mTORC1 stimulates the proliferation of fibroblasts and the synthesis of collagen; on the other hand, mTORC1 increases the expression of TGF-β1, which mediates the development of DN fibrosis (307, 308). Therefore, blocking the mTOR pathway can significantly increase the number of Treg cells, which promotes the improvement of diabetic nephropathy.

Although these studies suggest a possible link between Foxp3+ Treg cells and the progression of CKD disease, however, due to the complex etiology of CKD, the proportion of immune abnormalities is not known at present, and we only describe CKD from the perspective of abnormal metabolism of Treg cells. Further studies are still needed to understand the exact role of Treg in more targeted treatment plans.

#### The Role of Treg Cell Metabolism in Lupus Nephritis

SLE is a prototypic systemic autoimmune disease, as well as a multisystem heterogeneous disease (309). Immune abnormalities interact with various other factors, leading to a decrease in T lymphocytes and a decline in Treg cell function in SLE (310). LN is an autoimmune disease secondary to SLE, characterized by cell proliferation and immune complex deposition, accompanied by significant clinical manifestations of renal damage (311). Studies have demonstrated that the metabolic patterns of Th17 cells and Treg cells affect the balance of both cell types (312) (**Figure 5**). Th17 cells mainly rely on glycolysis to provide energy (313), while Treg cells mainly rely on fatty acid oxidation pathways to provide energy (314). Inhibition of glycolysis and fatty acid oxidation can promote the development and differentiation of Treg cells, and inhibit the differentiation of Th17 cells (315). Deficient or scarce Treg cells have been found both in murine models of SLE and in human SLE studies (316). Studies have shown that peripheral blood Treg cells decline in number and abnormal Treg cell phenotypes are present in SLE patients (317). Sirolimus has been shown to be an effective retention steroid for the treatment of renal and non-renal manifestations of SLE (318).

Cellular metabolism regulates the differentiation and function of T cells, thus participating in the initiation and progression of SLE inflammation. These characteristics are as follows:

(1) Mitochondrial hyperpolarization: T cells are chronically mobilized, and their mitochondria are hyperpolarized in SLE patients and lupus-prone mice (319, 320); thus, the high mitochondrial transmembrane potential will be expressed in Treg cells from SLE patients (138). The hyperpolarization of mitochondria affects the process of reducing oxygen to water by electron and proton transfer during oxidative phosphorylation, leading to increased oxygen consumption and ROS generation, thereby reducing energy synthesis (320, 321). ROS can oxidize proteins and cause DNA mutations, causing cell damage and cell aging, and excessive ROS will attack the protease complex on the oxidative respiratory chain, leading to mitochondrial dysfunction, reducing ATP production, inhibiting mTORC1, and promoting the differentiation of T cells into Treg cells (319, 320, 322), which is a vital cause of Treg cell functional deterioration. Furthermore, in lupus-susceptible mice, blocking of Rab geranylgeranyl transferase with Rab geranylgeranyl transferase inhibitor (3-PEHPC) could reverse dynamin-related protein 1 (Drp1) consumption, mitochondrial accumulation, and nephritis, confirming that HRES-1/Rab4 regulation of mitochondrial homeostasis is the pathogenesis and potential therapeutic target of SLE (323).
(2) Hyperactivated carbohydrate metabolism: Excessive activation of glucose metabolism leads to the accumulation of energy in T cells (324). Increased ATP content weakens AMP-AMPK signal transduction and then activates mTORC1 in SLE patients (325). The enhancement of mTORC1 activity could inhibit Treg cell activation and function (326).
(3) Enhanced lipid synthesis: Acetyl-CoA is produced by the β oxidation of fatty acids while cholesterol is generated *via* the catalysis of hydroxy methylglutaryl coenzyme A (HMG-CoA) reductase (327). The key enzyme ACC (acetyl-CoA carboxylase) that inhibits the FAS and cholesterol synthesis can also inhibit the expression of Th17 and promote the differentiation of Treg cells (131), thereby reducing the autoimmune response of SLE patients. Studies have shown that the synthesis of lipid rafts (including glycosphingolipids and cholesterol) in SLE patients is increased, and CD4+ T cells from active SLE patients have more lipid raft synthesis than CD4+ T cells from healthy individuals (328), which influences the proliferation and function of Treg cells. In particular, cholesterol biosynthesis was demonstrated mechanistically to be important in promoting Treg cell activation, proliferation, and function (329).
(4) Amino acid dysfunction: Catabolism of the amino acid tryptophan generates metabolic intermediates such as kynurenine that can bind the aryl hydrocarbon receptor on T cells and promote pTreg cell induction (182). The binding of CTLA4 on Treg cells to the costimulatory molecules CD80 and CD86 on antigen-presenting cells (APCs) induces amino acid-consuming enzyme (such as IDO and arginase 1) expression in Treg cells (330). The activities of these enzymes reduce the availability of amino acids (for example, tryptophan, arginine, histidine, and threonine) to surrounding T cells, inhibiting mTOR signaling *via* the lipid phosphatase PTEN and blocking the proliferation of Teff cells, thus promoting Treg cell induction (183).
(5) Highly activated mTOR pathway: High activation of the mTOR pathway may increase protein synthesis, leading to protein accumulation in Treg cells (331), enhancing cell metabolism, promoting the autophagy system of Treg cells, and leading to dysfunction and reduced differentiation of Treg cells (332, 333). A study demonstrated that mTORC2 plays a proinflammatory role in blocking Treg generation in SLE. mTORC2 can activate the Akt signaling pathway and promote glucose metabolism, while Treg cells are mainly powered by FAO (334). Therefore, the activation of mTORC2 will inhibit the development and differentiation of Treg cells, and mTORC2 blockade is important to lineage stabilization and functional maturation of Treg cells except for Treg cell differentiation. Additionally, Treg cells effects appear to be significantly modulated in humans compared to mice, which may be explained by the fact that blocking mTOR with rapamycin can complete nephritis blocking in several lupus-susceptible strains without affecting Treg cells in mice.

Dietary habits and nutritional factors can regulate Th17/Treg cell balance by affecting T cell metabolism. A balanced diet may help prevent and manage SLE. A low cholesterol diet could improve Th17/Treg cell balance by activating liver x receptor α and β (LXRs), which are nuclear receptors modulating cholesterol metabolism (335). High glucose intake can induce Th17 cells by upregulating mitochondrial ROS in T cells, thus enhancing self-immunity (336). Long-chain fatty acids enhance Th17 cell differentiation, whereas short-chain fatty acids derived from a fiber-rich diet expand Treg cells and reduce IL-17 production (315).

#### The Role of Treg Cell Metabolism in Other Kidney Diseases

In addition to the three kidney diseases mentioned above, there are also several related to Treg cell metabolisms, such as IgA nephropathy, glomerulonephritis, nephrotoxic serum nephritis, and renal cell carcinoma.

#### IgA Nephropathy

IgA nephropathy (IgAN) is an autoimmune disease, and its immune pathogenesis is a multilevel process (337). In patients with lgAN, the level of abnormally glycosylated circulating IgA increases, which induces the formation of autoantibodies of IgA and IgG and then forms a circulating immune complex of autoantibodies of IgA and lgG (338). These immune complexes contribute to mesangial cell proliferation and excessive production of extracellular matrix, cytokines, and chemokines, eventually leading to glomerular sclerosis (11, 19, 339–341).

In IgAN patients, serum soluble fms-like tyrosine kinase 1 (sFlt-1) levels are remarkably enhanced, and subsequently, sFlt-1 raises the mitochondrial membrane potential, facilitating mitochondrial-mediated apoptosis (342). A study has shown that patients with less histological injury and proteinuria have higher urinary mtDNA copy numbers, which suggests that mitochondrial damage occurs in the early stage of IgAN (209, 343).

A study showed that patients with IgAN have a higher prevalence of dyslipidemia (319). Excessive ox-LDL content will weaken the immunosuppressive function of Treg cells, and ox-LDL can induce apoptosis of Treg cells by activating P38/MAPK (344), mitochondria (345), and lysosome signaling pathways (**Figure 5**). In addition, ox-LDL can induce cells to produce endogenous ROS (340), which increases the production of lipid peroxides in Treg cells. The damage of oxidized lipids to cells leads to abnormal cell lipid metabolism and impaired exportability, thus inducing apoptosis of Treg cells (341, 346).

Some studies have shown that in rats with IgAN, p-mTOR and phosphorylation of p70 S6 kinase (P-S6K1) are upregulated, which indicates that the mTOR pathway is highly activated and participates in the development of IgAN (343, 347–350).

#### Glomerulonephritis

GN encompasses a wide variety of kidney diseases (351). Many GNs due to immunologically mediated glomerular damage result in renal dysfunction and proteinuria (352). Treg cells are essential for the autoimmune pathogenesis of GN in the kidney (353), as such, the activation of STAT3 in Treg cells induces the expression of CC chemokine receptor 6 (CCR6) on the cell surface (354) and promotes the transport of Treg cells to the inflammatory area of Th17 cells that also highly express CCR6 through the tight colocalization of CCR6 (355), thereby suppressing the immune response of pathogenic Th17 cells during the GN process. Treg cells can also use CC chemokine receptor 7 (CCR7) expressed on their own surface to migrate to the site of CCR7+ T cell activation and inhibit the activation of T cells (356). Treg cells with defective IL-10Ra expression can significantly reduce the production of IL-10 during GN, while Treg cells can significantly downregulate Th17 cells through IL-10 receptor signal transduction (357) (**Figure 5**). IL-10Ra is a key component that controls the immune function of Th17 cells in the GN process, and a large number of IL-10Ra-deficient T cells differentiate into Th17 cells, which aggravates the condition of GN (349). In addition, IL-6 stimulates Treg cells to produce cells, which have both pro-inflammatory and anti-inflammatory effects (329). ITreg cells can secrete the anti-inflammatory factors IL-10 and IL-35, as well as the pro-inflammatory factor IL-17 (350), and by inhibiting Th2 cells with anti-inflammatory effects, they mediate pro-inflammatory effects in GN (356). Treg cells also inhibit Th1 cells. Nosko et al. showed that Treg cells in which the transcription factor T-bet is activated enhance the ability of Treg cells to downregulate Th1 cell responses by inducing the expression of CXC chemokine receptor type 3 (CXCR3) (358). Studies have also reported that Treg cells inhibit GN driven by the Th1 immune response through the PD-1/programmed cell death-ligand 1 (PD-L1) pathway, and mediate renal protection (359) (**Figure 5**).

The kidney is rich in mitochondria, which meet its high energy demand through the oxidative phosphorylation process (360). Several studies have demonstrated that in glomerulonephritis nephropathy rats, the number of mitochondria in tubular epithelial cells is reduced and cristae structure is destroyed. Albumin and free fatty acid stimulation of cultured human tubular cells *in vitro* increased mitochondrial ROS, which led to mitochondrial damage (361, 362). Generating enough acetyl-CoA to feed into the Krebs cycle and then generating sufficient ATP through the mitochondria is an important process in Treg cells (363). Although the mechanism was not uncovered, the induction of Foxp3 in iTreg cells correlated with increased expression of mitochondria-associated genes (364) Mitochondrial dysfunction can cause abnormal metabolism of Treg cells, and the protective effect of Treg cells on the kidney is limited.

In addition to oxidative stress, GN can also undergo the deposition of lipid-associated molecules, including oxidized cholesterol, apolipoprotein (Apo), and ox-LDL. Oxidative and helix-related molecules accumulate on the glomerular basement membrane (GBM) along with other molecules (320, 365). ox-LDL can induce apoptosis of Treg cells by activating P38/MAPK (366). AMPK activity together with protein phosphatase 2A (PP2A) restrains the mammalian target of rapamycin complex 1 (mTORC1) signaling, thus promoting Foxp3 expression and the proliferation of Treg cells (333, 367).

In patients with little immune deposition glomerulonephritis, a large amount of the glomerulus and even more cells in the tubulointerstitial area express mTOR (368). Upon Treg cell activation, the increase in mTOR signaling upregulates interferon regulatory Factor 4 (IRF4) (367), which further promotes genes for cellular growth, glycolysis, OXPHOS, and fatty acid metabolism, among others (369). These data suggest that promoting mTORC1 activity can promote the activation and function of Treg cells and support glycolysis and the OXPHOS metabolic pathway (369).

#### Nephrotoxic Serum Nephritis

Nephrotoxic serum nephritis (NTSN) nephritis is a type of focal segmental glomerulosclerosis (FSGS) that occurs in many kinds of renal disease and ultimately leads to kidney inflammation and fibrosis (370). The histological features of nephrotoxic serum nephritis are the accumulation of macrophages, cholesterol, and cholesteryl esters, as well as the deposition of extracellular matrix in sclerotic glomeruli. The disease is characterized by rapid inflammation and infiltration of leukocytes in the kidneys (371). In a mouse model with NTSN, Treg cells are endogenous immunosuppressive cells that protect kidney tissues from inflammation-mediated damage (372).

Increased mitochondrial ROS generation and mitochondrial oxidative damage are present in the glomeruli of patients with nephrotoxic serum nephritis, inhibiting the protective effect of Treg cells on the kidney (373) so that the expression of CCR7 on Treg cells is downregulated and affects the recruitment of Treg cells to the lymph nodes of NTSN (374). ROS can change the expression of transient receptor potential cation channel, subfamily C, member 6 (TRPC6) protein, or TRPC6 channel activity in kidney cells, thereby regulating Ca2+ signal transduction and mediating podocyte apoptosis (375) (**Figure 5**). Research has shown that the knockout of TRPC6 plays a protective role in NTSN (376). The accumulation of ROS in mitochondria induces mitochondrial dysfunction and apoptosis, eventually causing glomerular disease (377), which includes nephrotoxic serum nephritis. In addition, experiments have shown that the NTSN of CCR7 knockout mice is more serious, and abundant inflammatory cell infiltration was observed (378).

#### Renal Cell Carcinoma

Renal cell carcinoma (RCC) is one of the most common tumors and arises from the renal parenchyma urinary tubular epithelial system (379). There are many pathological types of renal cell carcinoma, of which clear cell renal cell carcinoma (ccRCC) is the most common (380). Renal cancer cells have a vigorous metabolism competing with immune cells for nutrients, thereby changing the metabolic mode of immune cells, and subsequently affecting their function and differentiation (381). Furthermore, substances produced by renal cancer cells, such as lactic acid and ROS, can cause damage to immune cells and reduce their antitumor effect (382).

Deletion of the von Hippel-Lindau (VHL) tumor suppressor gene in renal cancer cells leads to the accumulation of HIF-1α and an increase in Clut2 expression, which promotes aerobic glycolysis in renal cancer cells and leads to metabolic reprogramming of renal cancer cells (383). This aerobic glycolysis mode of cancer cells is called the “Warburg effect” (382). Hypoxia-mediated expression of HIF-1α selectively upregulates the expression of inhibitory ligands, such as PD-L1, and promotes T cell immunosuppression (384). Such hypoxia-mediated changes also promote Treg cell differentiation and homeostasis (385). The propagation of kidney cancer cells is highly dependent on glycolysis (386), which affects the function of Th17 cells that also rely on glycolysis but does not affect the survival of Treg cells that depend on fatty acid oxidation.

The proliferation of renal cancer cells consumes a lot of glutamine and competes with the surrounding macrophages for glutamine in the extracellular matrix (387). This is related to the promotion of the expression of ASCT2 and SLCIA5 by MYC (388) in renal cancer cells, leading to a large amount of glutamine being transported into the cell, which in turn activates the PI3K-Akt-mTOR signaling pathway and promotes the metabolism of glutamine and the synthesis of protein (389). The metabolic waste products of amide will promote the differentiation of Treg cells (382). In addition, lactate can induce the secretion of IL-23 by macrophages infiltrated by tumor cells (169). IL-23 activates the JAK/STAT pathway of Treg cells, increases the phosphorylation of STAT3, activating the proliferation of Treg cells, promotes the expression of IL-10 and TGF-β, thus inhibiting the killing effect of TCL on renal cancer cells (390, 391) (**Figure 5**).

IDO is overexpressed in a variety of cancers (392–394). IDO activity reduces local tryptophan availability in the proximity of Treg cells (395). A low concentration of tryptophan activates a stress response pathway in Treg cells through the protein kinase general control nonderepressing-2, which inhibits mTORC2 and prevents it from phosphorylating Akt, plus contributes to the maintenance of Treg suppressive function (395–397).

A large amount of lipid accumulation is found in renal cell carcinoma. HIF-1α in renal cell carcinoma inhibits the activity of CPT1 on the outer mitochondrial membrane and prevents the β-oxidation of fatty acids (398), which is important for the differentiation of Treg cells, and its blockade could prevent the accumulation of this immunosuppressive population (199). AMPK in renal cancer cells, a sensor of nutrient deprivation and metabolic stress, is inactivated in the AMPK-GATA3-ECHS1 signaling pathway (399), inhibiting the expression of the transcription factor GATA3 and leading to a decrease in the synthesis of ECHS1. AMPK activation can promote the formation of Treg cells while reducing Th1 and Th17 cells (199), thus, leading to unwanted immune modulation in the context of RCC. In addition, the inactivation of AMPK reduces the activation of adipose triacylglyceride lipase (ATGL) and inhibits the decomposition of triglycerides into fatty acids, thereby inhibiting the catabolism of fatty acids; at the same time, the inactivation of AMPK weakens the inhibitory effect on acetyl-CoA carboxylase (ACC) and promotes the synthesis of fatty acids. Berod et al. showed that inhibition of ACC1 restrains the differentiation of Th17 cells and promotes the differentiation of anti-inflammatory Foxp3+ Treg cells (400, 401).

## Conclusion

The immune system searches for pathogens and other danger signals *in vivo* at all times. In recent years, the field linking immunity and metabolism has expanded rapidly (402). In renal diseases, T cells are involved in different abnormal metabolic pathways, such as increased oxidative stress, mitochondrial dysfunction, enhanced glycolysis, abnormal lipid synthesis, glutaminolysis, and highly activated mTOR, which all influence Treg cell proliferation and differentiation.

Inhibition of different metabolic pathways *via* drugs can modify Th17 cells to Treg cells. For example, inhibition of glycolysis (209, 403–405), lipid synthesis (328, 406, 407), and mTOR signaling (211, 408, 409) can control inflammation and alleviate disease activity in lupus mice and SLE patients (**Table 1**). Short-chain fatty acids, which are derived from a fiber-rich diet, can downregulate IL-17 production and amplify Treg cells (410–412). Treg cell metabolism therapy has great potential in many forms of renal diseases. Promoting the proliferation or function of Treg cells by mediating various metabolic pathways are also possible treatments in the future for multifarious diseases that affect the kidney.

It is important to note that many studies involving the immune metabolism of Treg cells have been based on model animal studies (mostly mice) or *in vitro* human cells. Pharmacological or genetic manipulation of metabolic processes in mouse models of human autoimmune diseases offers new opportunities to treat human diseases, but it is not clear how Treg cell immune metabolism is altered in many people with kidney disease. Therefore, we can make inferences based on published articles, but cannot be sure that these experimental results are consistent in animal and human kidney disease. This is a knowledge gap in Treg cell immune metabolism. Treg metabolism may become a target for future treatment of various kidney diseases.

## Author Contributions

ZH, KM, and HT were involved in the conception of the study. ZH, KM, LS, and QN were involved in writing the article. HL, JZ, XS, YL, MC, and CL critically revised the manuscript. All authors read and approved the final manuscript.

## Funding

The work was supported by the Foundation of Popularization project Department of Sichuan Health commission (19PJYY0731); The Foundation of Key R&D plan of Sichuan Province (2019YFS0538); and The project of 2020 High-level Overseas Chinese Talent Returning Funding.

## Conflict of Interest

The authors declare that the research was conducted in the absence of any commercial or financial relationships that could be construed as a potential conflict of interest.

## Publisher’s Note

All claims expressed in this article are solely those of the authors and do not necessarily represent those of their affiliated organizations, or those of the publisher, the editors and the reviewers. Any product that may be evaluated in this article, or claim that may be made by its manufacturer, is not guaranteed or endorsed by the publisher.
